# Evaluation of Five Host Inflammatory Biomarkers in Early Diagnosis of Ventilator-Associated Pneumonia in Critically Ill Children: A Prospective Single Center Cohort Study

**DOI:** 10.3390/antibiotics12050921

**Published:** 2023-05-17

**Authors:** Maria Sdougka, Maria Simitsopoulou, Elena Volakli, Asimina Violaki, Vivian Georgopoulou, Argiro Ftergioti, Emmanuel Roilides, Elias Iosifidis

**Affiliations:** 1 Pediatric Intensive Care Unit, Hippokration General Hospital, 54942 Thessaloniki, Greece; 2 Infectious Disease Unit, 3rd Department of Pediatrics, School of Medicine, Faculty of Health Sciences, Hippokration General Hospital, 54942 Thessaloniki, Greece; 3 Medical Imaging Department, Hippokration General Hospital, 54942 Thessaloniki, Greece

**Keywords:** ventilator-associated pneumonia, critically ill children, biomarkers

## Abstract

**Background:** Early diagnosis of ventilator-associated pneumonia (VAP) remains a challenge due to subjective clinical criteria and the low discriminative power of diagnostic tests. We assessed whether rapid molecular diagnostics in combination with Clinically Pulmonary Index Score (CPIS) scoring, microbiological surveillance and biomarker measurements of PTX-3, SP-D, s-TREM, PTX-3, IL-1β and IL-8 in the blood or lung could improve the accuracy of VAP diagnosis and follow-up in critically ill children. **Methods**: A prospective pragmatic study in a Pediatric Intensive Care Unit (PICU) was conducted on ventilated critically ill children divided into two groups: high and low suspicion of VAP according to modified Clinically Pulmonary Index Score (mCPIS). Blood and bronchial samples were collected on days 1, 3, 6 and 12 after event onset. Rapid diagnostics were used for pathogen identification and ELISA for PTX-3, SP-D, s-TREM, IL-1β and IL-8 measurements. **Results:** Among 20 enrolled patients, 12 had a high suspicion (mCPIS > 6), and 8 had a low suspicion of VAP (mCPIS < 6); 65% were male; and 35% had chronic disease. IL-1β levels at day 1 correlated significantly with the number of mechanical ventilation days (r_s_ = 0.67, *p* < 0.001) and the PICU stay (r = 0.66; *p* < 0.002). No significant differences were found in the levels of the other biomarkers between the two groups. Mortality was recorded in two patients with high VAP suspicion. **Conclusions:** PTX-3, SP-D, s-TREM, IL-1β and IL-8 biomarkers could not discriminate patients with a high or low suspicion of VAP diagnosis.

## 1. Introduction

Ventilator-associated pneumonia (VAP) is the second most common hospital-acquired infection after bloodstream infections in critically ill children [1]. Pneumonia development leads to longer duration of mechanical ventilation, prolonged hospital stay and broad-spectrum antibiotic use, and it contributes to high morbidity and mortality rates [2,3]. In addition, in critically ill children hospitalized with pneumonia, those with VAP have worse outcomes than those with severe community-acquired pneumonia [4].

Several pediatric studies report that the frequency of VAP in the Pediatric Intensive Care Units (PICUs) worldwide is in the range of 2 to 35%. Such significant variation is attributed to, among other things, differences in case definition, sampling procedure and diagnostic method [5,6]. The most recent National Health Safety Network (NHSN) module published by the Centers for Disease Control and Prevention on definitions specific to VAP underlines that early-onset VAP is suspected when microorganism invasion of the lower respiratory tract occurs on more than two consecutive calendar days from the date of event in patients on mechanical ventilator support [7]. However, due to the lack of universally employed diagnostic algorithms, an accurate VAP diagnosis remains a great challenge, hampering timely administration of antibiotic regimens, clear assessment of existing VAP burden in PICUs and development of effective preventive strategies [8].

The use of clinical scores such as the Clinical Pulmonary Infection Score (CPIS) (fever, leukocytosis, bronchial aspirates, oxygenation and radiographic pulmonary infiltrates) and microbiological tests are insufficient to discriminate VAP from other non-infectious conditions. This is due to the subjective assessment of clinical criteria, interobserver variability, inherently low specificity or sensitivity as well as delayed differential diagnosis ranging from 48 to 72 h [9]. Thus, in conjunction with clinical scoring for suspected VAP, the use of molecular diagnostic platforms for the rapid identification of the most common respiratory pathogens, combined with the use of biomarkers of infection employing non-invasive sampling procedures, needs to be explored to determine their clinical value in early diagnosis of VAP in critically ill children.

Accurate and rapid identification of true lung infection for targeted antibiotic treatment is the most important attribute that underscores the rationale for using biomarkers in clinical practice. Although the value and net health benefit for a number of biomarkers in VAP diagnosis has been investigated, the results of clinical studies remain contradictory. For example, the diagnostic value of single measurements performed for PCT (procalcitonin), a prohormone released in serum in response to inflammation, CRP (C-reactive Protein), an acute-phase protein and soluble triggering receptor expressed on myeloid cells and s-TREM biomarker, a glycoprotein member of the immunoglobulin family up-regulated in the presence of pathogens, has not been demonstrated as they cannot discriminate between suspected VAP and non-VAP cases [10,11,12]. SP-D (surfactant protein D, expressed by type II alveolar cells and involved in innate immunity on all mucosal surfaces, was found to be a bacterial species-specific differentiating factor in children with VAP. In VAP diagnosis, it was the most sensitive to PTX-3 (pentraxin-3), a member of the pentraxin subfamily correlated with lung injury severity in acute respiratory syndromes [13,14].

However, studies have shown that combining results from measurements of multiple biomarkers may provide significant discriminative power between infectious and non-infectious causes of inflammatory responses [15]. Systematic analyses concluded that a panel of biomarkers measured at different time points for grasping biomarker dynamics, used in conjunction with clinical diagnosis and scoring systems, may significantly improve early VAP diagnosis and antibiotic therapy [16,17].

Most of these biomarkers as well as cytokines such as IL-1β (interleukin-1-beta) and IL-8 (interleukin-8) have been investigated mostly in serum and bronchoalveolar lavage (BAL) samples to evaluate their association with VAP in adult patients. In current clinical practice, both CRP and PCT measurements are used in combination with clinical and microbiological criteria [17,18]. However, according to the most recent clinical guidelines for VAP diagnosis in adults, the initiation of antibiotic therapy should be driven by clinical criteria alone without taking into account CRP or PCT [19].

Unfortunately, there are even fewer data on the role of biomarkers for VAP diagnosis in children. The biomarkers s-TREM and PTX-3 have been investigated in only four pediatric studies with controversial results regarding their diagnostic accuracy [13,20,21,22], whereas no studies to date exist for the clinical value of IL-1β and IL-8 in early diagnosis of VAP in critically ill children.

In this study we assess whether using a polymerase chain reaction (PCR)-based rapid diagnostic tool to detect VAP-associated pathogens and resistance genes in the lower respiratory tract, in combination with CPIS scoring, microbiological tests and the levels of PTX-3, SP-D, s-TREM, IL-1β, and IL-8 in serum and/or lower respiratory tract aspirate, could improve the accuracy of VAP diagnosis and follow-up in critically-ill children.

## 2. Results

*Characteristics of the patients:* Over the 16-month study, 27 children were screened and 20 (74%) were included in the study. According to the mCPIS, the high VAP suspicion group (mCPIS > 6) was made up of 12 children and the low VAP suspicion group (mCPIS < 6) consisted of 8 children. Of the total population, 65% were male. The median age was 24.5 (6–141) months in the high suspicion group and 129 (28–184) months in low-suspicion group. Thirty five percent had chronic disease and 40% acute illness (Table 1). All children were on mechanical ventilation and were treated with antibiotics on day 1 of the study. Analysis of clinical characteristics presented in Table 1 showed no significant differences between high and low VAP suspicion groups.

**Figure 1:**
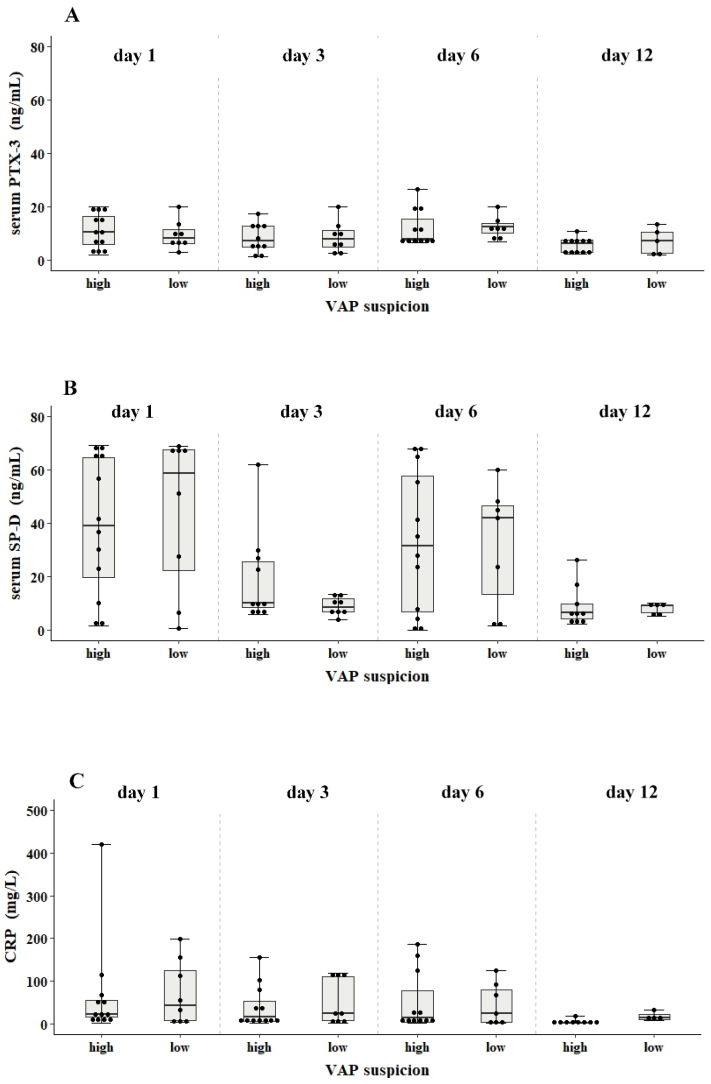
Serum PTX-3, SP-D and CRP levels of critically ill children with VAP suspicion. Box (interquartile) and whisker (range) plots show the concentration of PTX-3 (ng/mL; panel (**A**)), SP-D (ng/mL; panel (**B**)) and CRP (mg/L; panel (**C**)) in blood at days 1, 3, 6 and 12 from patients with mechanical ventilation. The patients with VAP suspicion were assigned into two groups based on CPIS scores: high (CPIS > 6) and low (CPIS < 6). Statistically significant differences between groups were examined using the non-parametric ANOVA Kruskal–Wallis with Dunn’s multiple comparisons test.

*Molecular and microbiological assessment*: Rapid molecular diagnostics conducted on bronchial secretions of patients obtained on day 1 identified *Staphylococcus aureus* in one patient and *Acinetobacter baumannii* in three others, all of whom were in the low VAP suspicion group. Blood cultures during the four timepoints of the study remained negative. Two non-colonized patients in the low VAP suspicion group, with declining CRP levels on day 6 (91 and 24 mg/L), developed sepsis on day 12 of the study, which increased CRP levels to 278 mg/L. These two patients were therefore excluded from the data analysis of the CRP biomarker on day 12 of the study (Figure 1, panel C).

**Figure 2:**
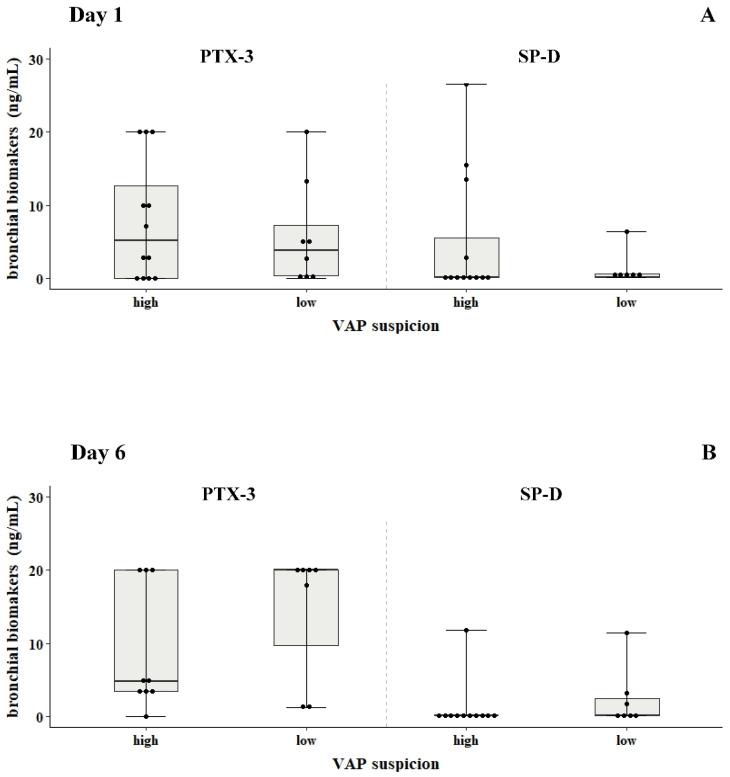

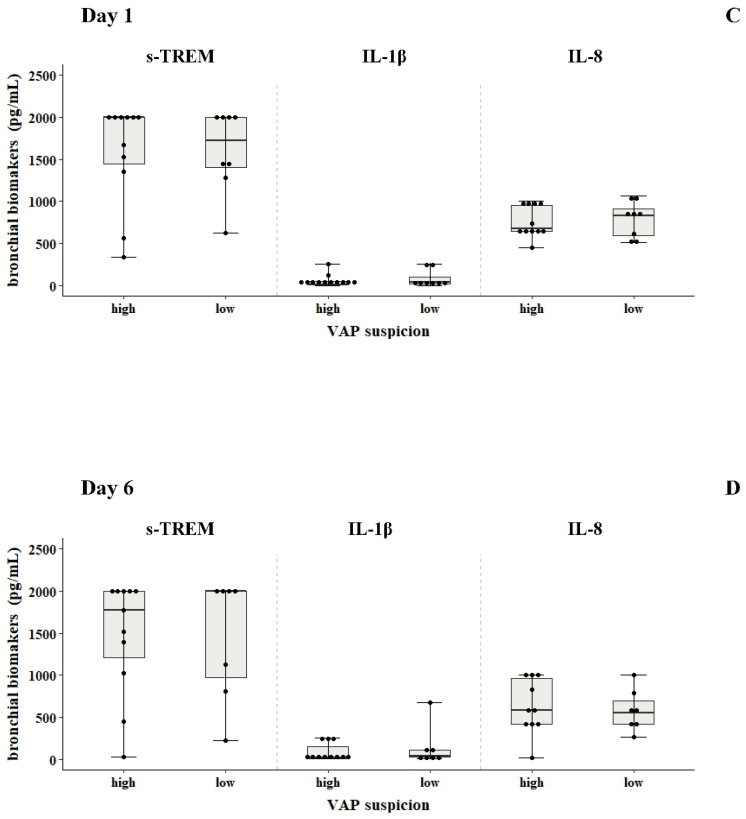
PTX-3, SP-D, s-TREM, IL-1β and IL-8 levels in bronchial aspirates of critically ill children with VAP suspicion. Concentrations of PTX-3 (ng/mL), SP-D (ng/mL), s-TREM (pg/mL), IL-1β (pg/mL) and IL-8 (pg/mL) in bronchial aspirates from patients with VAP suspicion on a mechanical ventilator at days 1 and 6 are shown (panels (**A**–**D**)). Statistically significant differences between the groups were examined using the non-parametric Kruskal-Wallis ANOVA test with Dunn’s multiple comparisons test.

*Biomarker levels in bronchial secretions and serum*: No significant differences were found in PTX-3, SP-D, s-TREM, IL-1β and IL-8 levels between the high and low VAP suspicion groups. In serum, the median levels of PTX-3, SP-D and CRP levels at the four timepoints in the high VAP group were 6–10 ng/mL, 6–39 ng/mL and 2–30 mg/L compared to 7–8 ng/mL, 8.3–58 ng/mL and 15–43 mg/L in the low VAP group, respectively (Figure 1, panels A–C). In bronchial aspirates, the levels of PTX-3, SP-D, s-TREM, IL-1β and IL-8 at days 3 and 6 were also similar in both VAP groups (Figure 2, panels A–D).

**Figure 3:**
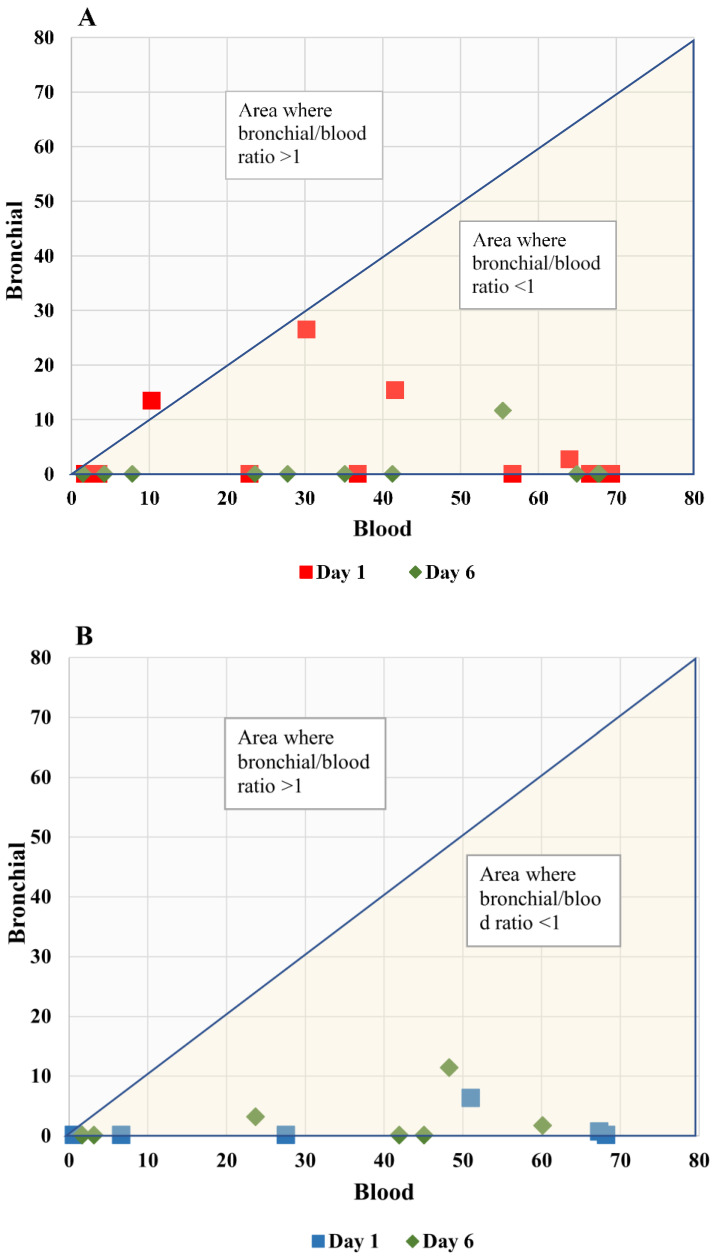
Bronchial vs. blood SP-D levels of critically ill children with high and low VAP suspicion. The SP-D level ratio between bronchial and blood samples on days 1 and 6 are shown for critically ill children with high (panel (**A**)) and low (panel (**B**)) VAP suspicion. The correlation coefficient between blood and bronchial SP-D levels (ng/mL) for patients with high VAP suspicion on day 1 (red square) was r = −0.133 (*p* = 0.68) and on day 6 (green diamond), r = 0.2 (*p* = 0.555). The correlation coefficient between blood and bronchial SP-D levels for patients with low VAP suspicion on day 1 (blue square) was r = 0.37 (*p* = 0.46) and on day 6 (green diamond), r = 0.51 (*p* = 0.24).

**Figure 4:**
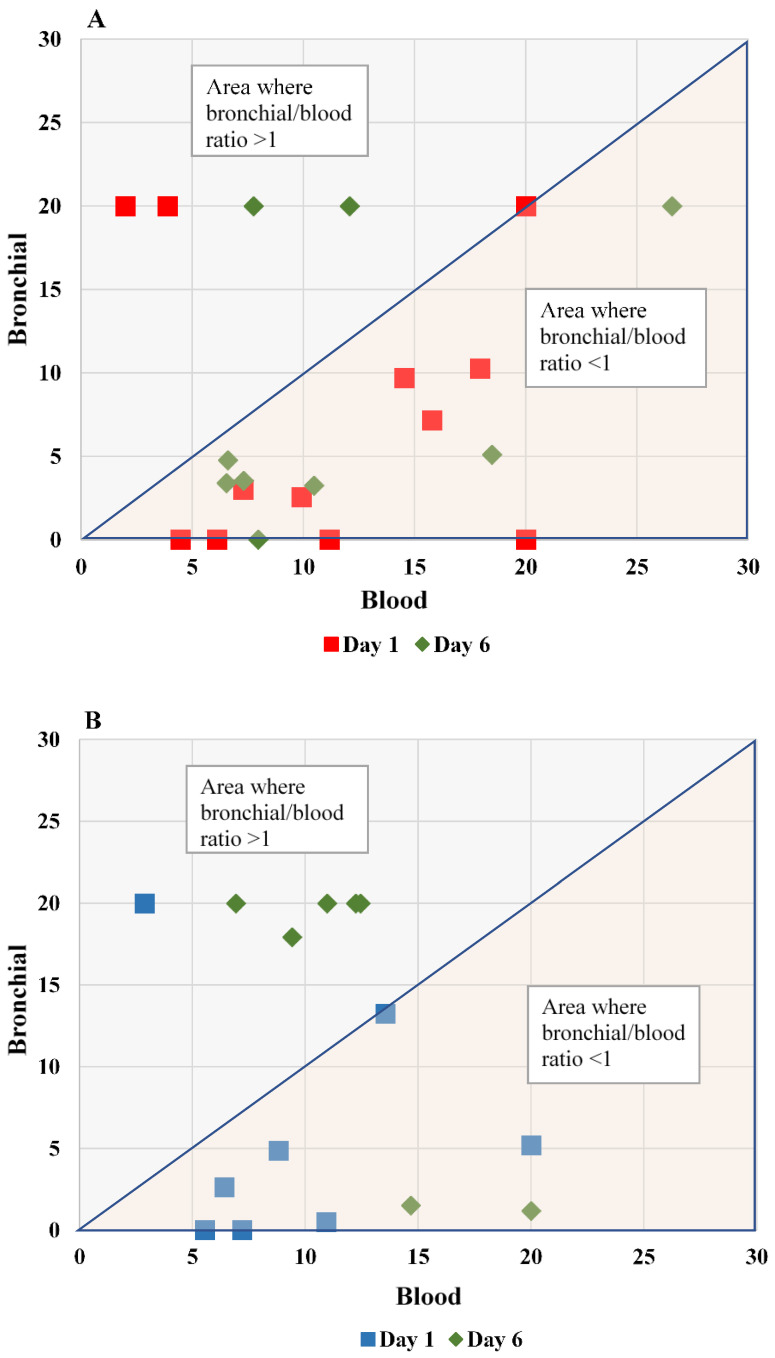
Bronchial vs. blood PTX-3 levels of critically ill children with high and low VAP suspicion. The PTX-3 level ratio between bronchial and blood samples in days 1 and 6 are shown for critically ill children with high (panel (**A**)) and low (panel (**B**)) VAP suspicion. The correlation coefficient between blood and bronchial PTX-3 levels (ng/mL) for patients with high VAP suspicion on day 1 (red square) was r = −0.048 (*p* = 0.88) and on day 6 (green diamond), r = 0.42 (*p* = 0.25). The correlation coefficient between blood and bronchial PTX-3 levels for patients with low VAP suspicion on day 1 (blue square) was r = 0.16 (*p* = 0.69) and on day 6 (green diamond), r = −0.61 (*p* = 0.145).

*Correlation between blood and bronchial levels of biomarkers*: At patient level, no significant correlation was found between blood and bronchial SP-D levels for patients with high VAP suspicion on day 1 (r = −0.133; *p* = 0.68) and on day 6 (r = 0.2; *p* = 0.555): Figure 3, panel A). In addition, for patients with low VAP suspicion also there was no correlation between the levels of blood and bronchial SP-D levels on day 1 (r = 0.37; *p* = 0.46) or day 6 (r = 0.51; *p* = 0.24): Figure 3, panel B. Similarly, no correlation was found between blood and bronchial PTX-3 levels in the high VAP suspicion group on day 1 (r = −0.048; *p* = 0.88) or day 6 (r = 0.42; *p* = 0.25): Figure 4, panel A. In the low VAP suspicion group r = 0.16 for day 1 (*p* = 0.69) and for day 6 r = −0.61 (*p* = 0.145): Figure 4, panel B.

*Correlation between the level of biomarkers and patient outcomes*: Of all the biomarkers tested in this study (blood and bronchial), only the levels of IL-1β obtained on day 1 after enrollment correlated significantly with the number of mechanical ventilation days (r= 0.67; *p* < 0.001) and PICU stay (r = 0.66; *p* < 0.002).

Mortality was recorded for 2/20 patients only in the high VAP suspicion group (*p* = 0.49). One death happened 30 days after ICU discharge, whereas the other death occurred on day 3 of the study following a severe sepsis episode.

## 3. Discussion

In this prospective, pragmatic study conducted under real-life routine clinical practice conditions on mechanically ventilated critically ill children with suspicion of ventilator-associated pneumonia, biomarkers in the blood and bronchial aspirates of patients with a high or low suspicion of VAP diagnosis could not be discriminated. However, both the length of mechanical ventilation and ICU stay correlated significantly with the levels of IL-1β measured in bronchial aspirates on day 1 of VAP suspicion.

A gold standard for VAP diagnosis is still missing [6,19], and this has important implications for research into the epidemiology, natural history, treatment and prevention of VAP, especially in children. Although there is a shift toward more objective criteria in VAP diagnosis using the current ventilator-associated event (VAE) definitions, the new algorithm was developed mostly for epidemiological issues, not for clinical use [23]. Most research on VAP in children has been based on radiological definitions such as CDC pneumonia criteria and the CPIS [6]. A number of studies have shown the sensitivity and specificity of these definitions, including the mCPIS, for children [24,25,26,27]. In this study, children were enrolled with a different level of VAP suspicion, thereby avoiding the use of a specific definition that would bias our result and discriminate patients according to predefined criteria in the absence of a gold-standard definition.

As research into the implementation of fast-track diagnostics using syndromic panels grows, especially in ICU settings, it has been shown that molecular platforms have the potential to improve antimicrobial use and benefit patient outcomes compared to standard culture methods [28]. In our study, we used both bronchial aspirate cultures to identify and monitor detected bacteria as other studies have done, but we also employed rapid syndromic molecular diagnostics to identify potential pathogens such as viruses, non-culturable microorganisms and antimicrobial resistance profiles. Most of our patients had a negative molecular test, suggesting that VAP suspicion was clinically rather than microbiologically driven.

CRP is synthesized in the liver in response to the increased release of inflammatory cytokines at the site of the disease. Although its role in diagnostics includes a delay in responding to clinical stimuli and poor specificity––as increased CRP levels are found in a variety of pathologies other than VAP––studies have shown that it is a robust biomarker for acute-phase conditions [29]. In our study, two non-colonized patients of the low VAP suspicion group with declining CRP levels developed sepsis, increasing CRP levels at least threefold between the two monitored timepoints. Large clinical studies conducted with adult populations demonstrated its clinical value in hospital admissions because increased serum CRP levels have been significantly associated with increased 30-day mortality and need for mechanical ventilation [30], whereas monitoring CRP levels often seems to be useful for the early prediction of VAP [31]. However, the clinical value of CRP levels combined with PRISMIII to predict early VAP diagnosis in the pediatric population seems to be limited [32]. In our study, we also found no significant differences in the CRP serum levels measured at four timepoints between the high and low VAP groups. As the diagnostic accuracy may have been affected by the small sample size, the next step would be to enlarge the sample size and monitor CRP levels daily to evaluate the clinical utility of CRP in early VAP diagnosis.

This is the first time that PTX-3 levels had been evaluated simultaneously and repeatedly in both bronchial aspirates and serum in children with VAP suspicion. Tekerek et al. found that PTX-3 serum levels were significantly higher in pediatric patients with microbiologically confirmed VAP compared to children with suspected VAP and controls, where an optimal cut-off value for PTX-3 in serum was reported to be 4.19 ng/mL. The mCPIS was used for VAP diagnosis [13]. In our study, using the mCPIS score for patient classification, we found that the majority of patients with high and low suspicion of VAP had PTX-3 serum levels above the aforementioned cut-off value and the difference between the two groups was not significant. This could have been attributed to a different case mix but also to subjective limitations seen in mCPIS [33]. In addition, we found no correlation between the blood and bronchial levels of PTX-3 in patients with high or low VAP suspicion on day 1 or day 6. Two other studies conducted in adult patients with VAP found a cut-off value for PTX-3 in serum to be 16.43 and 2.56 ng/mL [34,35]. Only in one of these studies were serum PTX-3 levels measured sequentially starting from the day of intubation [34] In our pragmatic study, serial measurements of PTX-3 levels in serum remained elevated (6–10 ng/mL) among children with high and low suspicion of VAP probably because of other factors that may have influenced these levels.

The measurement of SP-D serum levels had been evaluated previously in one study and were proven to be the most sensitive biomarker for VAP diagnosis in critically ill children [13]. The cut-off value for this study was found to be 137.25 ng/mL, which was too high for our study population. All of our patients with either high or low VAP suspicion had a much lower SP-D level (6–42 ng/mL) during all four sequential serum measurements within the 12-day interval after VAP suspicion and study enrollment. Such value diversity calls for the validation of the optimal cut-off values for numerous serum biomarkers, including SP-D and PTX-3, in multicenter cohort studies of ventilated pediatric patients using a standard methodology.

The use of biomarkers in lower respiratory tract samples is attractive for most physicians and has been explored in many adult and a few pediatric studies [6,13,36,37]. In our study, five biomarkers––s-TREM, SP-D, PTX-3, IL-1β and IL-8––were for the first time simultaneously measured in bronchial aspirates of critically ill children with suspected VAP at two timepoints. Among these biomarkers, s-TREM was evaluated in four studies, three pediatric [20,21,22] and one neonatal [38], with conflicting results concerning the cut-off values of s-TREM for VAP diagnosis. Similarly, SP-D levels in BAL were evaluated in two pediatric studies [14,22] with conflicting conclusions regarding the clinical value of this biomarker. Specifically, one study concluded that SP-D has poor discriminatory power between VAP and colonization [22], whereas the second reported that elevated BAL SP-D levels represented a robust indication for a presumed nosocomial inoculation [14]. In addition, our study found no correlation between the levels of SP-D in bronchial aspirates and blood for either patient group. This indicated that using both bronchial and serum levels may not have helped to discriminate patients with or without VAP and that more data are needed to evaluate the role (if any) and the corresponding cut-off values of SP-D in children with VAP.

The inflammatory mediators IL-8 and IL-1β have been evaluated only in adult patients as part of a panel of biomarkers having the potential to correctly classify VAP cases from patients with brain injury or ventilated patients with non-pulmonary sepsis [39,40]. The authors concluded that patients who developed VAP had increased levels of these biomarkers, reflecting an inflammatory response to infection without however being able to differentiate VAP pneumonia in patients with brain injury or non-pulmonary sepsis. Nevertheless, a prospective multicenter study in 12 adult ICUs showed that low concentrations of IL-1β and IL-8 in BAL samples can confidently exclude VAP [36]. In our study, although none of these biomarkers had a predictive role for VAP diagnosis, IL-1β levels on day 1 were associated with mechanical ventilation and ICU stay. The use of endpoint clinical characteristics such as morbidity and mortality may have a more predictive, prognostic role as well as an added value for the patient besides exploring the validity of a current or new VAP algorithm [23,41]. Combining existing VAP diagnostic modules (including biomarkers) and exploring the best association with patient outcomes may be the best way to identify potential modifiable factors that would improve quality improvement in ventilated critically ill children [23]. However, this needs to be supported by multi-center and large-scale studies in children.

The strengths include rigorous inclusion criteria, collection of detailed information on standard of care testing and clinical outcomes as well as detailed assessment of both microbiological and molecular specimen testing for patient enrollment. The limitations of this study are the small number of patients and the antibiotic administration to all patients during the study enrollment. The latter could have led to borderline cases that influenced the overall expression levels of biomarkers in bronchial aspirates or blood. The fact that the bronchial/blood ratios were found to be non-discriminatory could have been due to the unequal compartmentalization of PTX-3 and SP-D at the time of sample collection. Although the children in this study were classified on the basis of VAP suspicion using the mCPIS score, the subjectivity and sensitivity of the score has been criticized [33]. However, it has been used in most studies exploring VAP diagnosis both in children and adults as well as in clinical practice [6]. The potentially missing of culture-based identified VAP pathogens was minimized by implementing multiplex PCR-based syndromic panel diagnostics. In addition, viral VAP, although rare, has clinical features that cannot be easily differentiated from bacterial infection [42].

In conclusion, the results of this study, supported by the existing literature, so far show that the clinical value of using biomarkers to diagnose VAP in critically ill children remains suggestive. The implementation of molecular diagnostics using syndromic panels to detect VAP-associated pathogens, especially in ICU settings, seems to benefit patient outcomes compared to standard culture methods. There is an urgent need for large multicenter cohort studies to set the baseline levels of candidate biomarkers to minimize selection bias and focus on outcomes such as morbidity and mortality.

## 4. Materials and Methods

*Study design and Patient population*: This was a single-center prospective pilot cohort study conducted from March 2021 to December 2022 on mechanically ventilated critically ill children in an 8-bed multivalent PICU of a tertiary university-affiliated hospital. Children between 1 month and 14 years of age with clinical suspicion of VAP and on mechanical ventilation for at least 48 h were eligible for enrollment, after their parents or guardians signed an informed consent form. To reflect an typical real-life scenario in pediatric critical care, enrollment was based on three tailor-made ICU criteria for VAP suspicion: (1) purulent respiratory or positive bronchial aspirate culture and the initiation of antimicrobial agent(s) for suspicion of VAP infection according to local practice; (2) increased oxygen requirement (defined as >20%) and fever, hypothermia, leukocytosis or leukopenia, and the initiation of antimicrobial agent(s); and (3) radiological findings of new lung infiltrates and at least 2 criteria from the following: fever (>38 °C) or hypothermia (<36 °C), increase in oxygen requirement >20%, purulent respiratory secretions, white blood cells count <4 or >12 × 10^9^ cells/L and CRP > 10 mg/L. Patients were excluded if (1) informed consent was declined, (2) the patient was unlikely to survive 48 h after enrollment, (3) pregnancy had occurred for adolescent female subjects, and (4) if body weight was less than 3 kg. On day 1 of the study for all enrolled patients, the modified CPIS (mCPIS) tool for VAP diagnosis was used [33] to assign them to one of two groups: a high VAP suspicion (mCPIS > 6) and a low VAP suspicion (mCPIS < 6).

*Data and sample collection*: Patient data recorded on standard electronic case report forms included demographic, clinical, chest radiograph and culture data. PRISMIII scoring was used for mortality risk assessment and the mCPIS score for VAP diagnosis and management (Table 1). Blood samples for hematological and biochemical measurements were collected at 4 time points corresponding to days 1, 3, 6 and 12 after the onset of event. Bronchial samples collected by expert PICU practitioners were liquefied in a sterile 0.9% saline solution and divided into three aliquots: two were processed immediately for microbiological and molecular diagnostics analysis, and the other was frozen at −75 °C for biomarker assay measurements. Bronchial aliquots were cultured 30 min after collection.

*Culture procedures*: The collected samples were inoculated on blood and MacConkey agar by using sterile inoculating loop. After incubation at 37 °C for 24–48 h, bacterial growth was measured and colony counts ≥10^4^ CFU/mL were considered as the diagnostic threshold for infection. Species identification and susceptibility testing were determined by conventional means using the VITEK-2 automated system (bioMérieux, Marcy l’ Étoile, France).

*Molecular diagnostics analysis*: For rapid molecular diagnostics, bronchial aliquots were analyzed using the Biofire Filmarray pneumonia plus panel (bioMérieux) run on a multiplexed PCR-based diagnostic platform to test for the presence of 27 of the most common pathogens involved in lower respiratory tract infections and to identify antibiotic resistance markers: *bla_CTX-M_*, *bla_KPC_*, *bla_NDM_*, *bla_VIM_*, *bla_OXA48_*_-like_ and *Mec*A/*Mec*C genes. The cutoff value for colonization or infection was set at 10^4^ copies/mL.

*Biomarker analysis*: Serum and bronchial samples obtained at the four time points were frozen at −75 °C until processed by a sandwich ELISA assay following the manufacturer’s recommendations (Proteintech Group, Manchester, UK). The samples were diluted (four-fold for PTX-3 detection and two-fold for s-TREM, IL-1β and IL-8 measurements) and run in duplicate on a 96-well format. Final protein concentrations were obtained by being multiplied by the respective dilution factors. The range of detection for each protein tested was: 0.027–20 ng/mL for PTX-3, 31.25–2000 pg/mL for s-TREM, 3.9–250 pg/mL for IL-1 β and 15.6–1000 pg/mL for IL-8. The highest and lowest limits of each protein standard were used as sample values for the samples read as outliers by the assay. Protein concentrations for each biomarker and experimental condition were obtained using a four-parameter logistic regression curve fit.

*Statistical analysis*: Continuous variables were presented as mean ± SD and comparisons between the groups were determined using an unpaired Student’s *t* or a Mann–Whitney U test depending on data distribution. Categorical values were expressed as percentages and comparisons were made using chi square or Fisher’s exact tests.

A Spearman’s correlation coefficient was used for correlation (a) between the levels of two biomarkers (PTX-3 and SPD) in blood and bronchial aspirates in patient for both high- and low-risk VAP groups for the same day, and (b) between the level of biomarkers (in blood or bronchial aspirates) and patient outcomes (length of mechanical ventilation, length of ICU stay, length of hospital stay, ICU and hospital mortality).

All data analysis was performed using the statistics program Instat (GraphPad, Inc., San Diego, CA, USA) and IBM SPSS v28 software package. A two-tailed *p* value < 0.05 was considered statistically significant.

## Figures and Tables

**Table 1:** Clinical characteristics of the study population.

	Total Population	Patient Groups with VAP ^1^ Suspicion		*p* Value
	n = 20	High (n = 12)	Low (n = 8)	
sex, male, n (%)	13 (65)	9 (75)	5 (63)	
Age ^2^, mo	93 (6–184)	24.5 (6–141)	129 (28–184)	
underlying disease				
trauma, n (% )	3 (15 )	3 (25)	0.0	
surgery, n (% )	2 (10)	2 (17)	0.0	
chronic disease *, n (%)	7 (35)	3 (25)	4 (50)	
acute illness, n (%)	8 (40)	4 (33.3)	4 (50)	
PRISM ^3^ ΙΙΙ score ^2^	11 (5–27)	13 (5–27)	10.5 (5–19)	0.443 ^a^
body temperature, (°C) ^6^	37.7 (0.97)	37.9 (1.07)	37.3 (0.64)	
vasopressors/shock, n (%)	14 (70)	10 (83)	5 (63)	
transfusions, n (%)	7 (35)	5 (42)	2 (25)	
CVC ^7^, n (%)	20 (100)	12 (100)	8 (100)	
nasogastric tube, n (%)	20 (100)	12 (100)	8 (100)	
folley, n (%)	19 (95)	11 (92)	8 (100)	
enteral, n (%)	18 (90)	10 (83)	8 (100)	
antibiotics, n (%)	20 (100)	12 (100)	8 (100)	
parenteral nutrition, n (%)	2 (10)	2 (17)	0.0	
time to enrollment ^2^, d	6 (4–29)	6 (4–29)	7 (2–26)	0.6 ^a^
mCPIS ^2,5^	6.5 (3–9)	7.25 (5–9)	4.25 (3–8)	<0.01 ᵇ
positive culture, n (%)	4 (20)	0.0	4 (100)	
time on mechanical ventilation ^2^, d	29 (5–62)	21.5 (8–62)	32 (5–43)	0.716 ^a^
length of PICU stay ^2,4^, d	31.5 (7–62)	26.5 (7–62)	35.5 (7–55)	0.967 ^a^
length of hospital stay ^2^, d	56.5 (6–181)	56.5 (7–181)	65 (6–155)	0.53 ^a^
death in PICU, n (%)	1 (5)	1 (8)	0.0	
mortality, n (%)	2 (10)	2 (17)	0.0	0.49

^1^ VAP: Ventilator-Associated Pneumonia, ^2^ median, range, ^3^ PRISM: Pediatric Risk of Mortality, ^4^ PICU: Pediatric Intensive Care Unit, ^5^ mCPIS: modified Clinical Pulmonary Infection Score ^6^ mean ± standard deviation, ^7^ CVC: Central Venous Catheter. ^a^ Mann-Whitney U test, ^b^ Student’s *t* test, * GABA transaminase deficiency, pantothenate kinase-associated neurodegeneration, Batten syndrome, cerebral palsy, epileptic encephalopathy, Noonan syndrome, Leigh syndrome.

## Data Availability

We confirm that the data supporting the findings of this study are available on reasonable request.
